# Effect of β-blocker on clinical outcomes in patients with traumatic brain injury: a retrospective propensity-matched study

**DOI:** 10.3389/fphar.2025.1465657

**Published:** 2025-01-22

**Authors:** Yaxin Zhang, Tingting Liu, Wenwen Ji, Guangdong Wang

**Affiliations:** ^1^ Department of Neurology, Xiamen Humanity Hospital, Fujian Medical University, Xiamen, Fujian, China; ^2^ Department of Respiratory and Critical Care Medicine, First Affiliated Hospital of Xi’an Jiaotong University, Xi’an, Shanxi, China

**Keywords:** β-blocker therapy, traumatic brain injury, mortality, propensity score matching, clinical outcomes

## Abstract

**Background:**

Traumatic brain injury (TBI) represents a significant public health challenge due to its complex management. β-blockers may offer neuroprotective benefits, but their impact on TBI outcomes remains unclear. This study aims to evaluate the effect of β-blocker use on clinical outcomes in TBI patients.

**Methods:**

This retrospective cohort study included adult TBI patients, categorized into β-blocker and non-β-blocker groups. Propensity score matching (PSM) was utilized to balance baseline characteristics. Mortality was assessed through the application of multivariable Cox regression models and Kaplan–Meier survival curves. Subgroup analyses examined the consistency of the results.

**Results:**

A total of 1,516 patients were included in the study, with 750 receiving β-blocker therapy and 766 not receiving it. After PSM, 473 pairs of patients were matched. The analysis indicated that β-blockers significantly reduce 28-day mortality (HR 0.43, 95% CI: 0.31–0.60, P < 0.001). However, patients receiving β-blocker had considerably longer hospital stays (7.89 days vs. 5.45 days, P < 0.001) and ICU stays (2.94 days vs. 2.33 days, P < 0.001).

**Conclusion:**

β-blocker therapy is associated with improved short-term outcomes in patients with TBI, particularly in those with mild (GCS 13–15) and severe (GCS 3–8) TBI. However, no significant benefit was observed in patients with moderate TBI (GCS 9–12). This therapy may also prolong hospital and ICU stays.

## Introduction

Traumatic brain injury (TBI) represents a significant public health challenge due to its complex management. Nearly 50% of trauma-related deaths are attributable to head injuries, which also entail substantial morbidity and economic burden (Crupi et al., 2020; Thapa et al., 2021). The management of TBI remains challenging due to the complex pathophysiological processes involved, including primary injury from mechanical forces and secondary injury from biochemical cascades that exacerbate neuronal damage (Crupi et al., 2020; Thapa et al., 2021). Current therapeutic strategies primarily focus on minimizing secondary injury through intracranial pressure management, surgical intervention, and supportive care (Crupi et al., 2020; Hawryluk et al., 2020). Despite these efforts, the mortality rate for severe TBI remains high, underscoring the need for novel therapeutic approaches (Maas et al., 2022).

The potential neuroprotective effects of β-blockers in TBI have garnered interest due to their ability to mitigate the catecholamine surge associated with TBI, which includes hypertension, tachycardia, and increased metabolic demand (Bouma and Muizelaar, 1992; Khalili et al., 2020). This catecholamine surge can exacerbate brain injury and contribute to poorer outcomes. Consequently, β-blockers might improve clinical outcomes by controlling these physiological responses.

Previous studies on β-blocker use in TBI patients have shown promising results, suggesting a potential reduction in mortality and improved outcomes (Ko et al., 2016; Zangbar et al., 2016; Ahl et al., 2017; Khalili et al., 2020). However, these studies frequently faced challenges such as limited sample sizes and inconsistent baseline characteristics, which impacted the reliability and applicability of their conclusions. To address these limitations, propensity score matching (PSM) can be employed. This method minimizes baseline differences between groups and approximates the effects of randomization in observational studies, thereby enhancing the validity and robustness of the findings (Jupiter, 2017).

In this study, our objective is to investigate the relationship between β-blocker therapy and clinical outcomes in TBI patients by using a large cohort from the Medical Information Mart for Intensive Care (MIMIC)-IV database.

## Methods

### Data source

Data for this study were sourced from the MIMIC-IV database, a large-scale, publicly accessible repository maintained collaboratively by the Massachusetts Institute of Technology and Beth Israel Deaconess Medical Center. MIMIC-IV includes detailed clinical information from over 70,000 patients admitted to intensive care units (ICU) (Johnson et al., 2023). The database provides extensive records, including physiological parameters, treatments, laboratory test results, and clinical notes, covering the entire trajectory from admission to discharge. The corresponding author, Guangdong Wang, has passed the assessment and obtained data usage permissions.

### Participant selection

**FIGURE 1:**
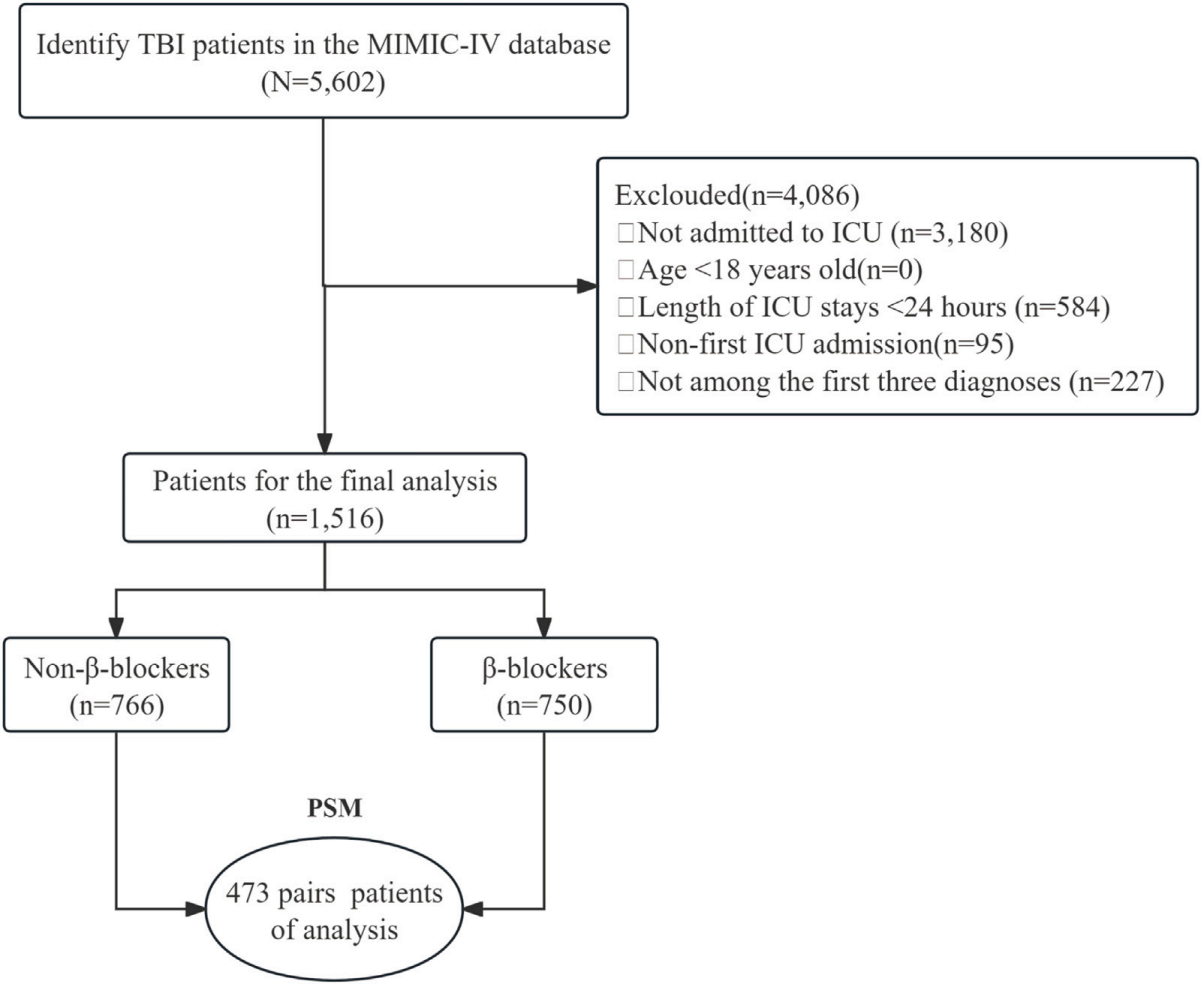
The flow chart of the study. TBI, traumatic brain injury; MIMIC-Ⅳ, Medical Information Mart for Intensive Care Ⅳ; ICU, intensive care unit; PSM, propensity-score matching.

Patients with TBI were selected using ICD-9 (code 85) and ICD-10 (code S06) codes. From an initial cohort of 5,602 patients, those who were not admitted to the ICU (n = 3,180), younger than 18 years (n = 0), had an ICU stay <24 h (n = 584), had a non-first ICU admission (n = 95), or did not have the TBI listed among the first three diagnoses (n = 227) were excluded. This led to a final cohort of 1,516 patients available for analysis. These patients were divided into two groups: 766 patients who did not receive β-blocker and 750 patients who did. PSM was applied, yielding 473 pairs of patients for the final analysis (Figure 1).

### β-blockers exposure

Exposure to β-blockers was defined as the administration of any β-blocker within the first 3 days after ICU admission, regardless of drug type, dosage, or pharmacokinetics. The β-blockers included in this study were metoprolol, atenolol, propranolol, esmolol, nadolol, bisoprolol, betaxolol, and acebutolol. These medications were administered either through intravenous push or orally. All β-blockers were treated equally to provide an overall assessment of β-blocker therapy’s effect on TBI outcomes.

### Variables

Data was collected within the first 24 h following ICU admission. The demographic variables included age and gender distribution (female and male), as well as racial composition (other and white). Vital signs and laboratory values gathered included heart rate, mean blood pressure (MBP), respiratory rate, oxygen saturation (SpO2), white blood cell (WBC), blood urea nitrogen (BUN), serum creatinine, sodium, potassium, and coagulation parameters. Additionally, we assessed comorbidities and clinical scores such as chronic heart failure (CHF), hypertension, diabetes, renal disease, Charlson Comorbidity Index, Glasgow Coma Scale (GCS), Sequential Organ Failure Assessment (SOFA) score, and Acute Physiology Score III (APS III). Interventions considered in the study included mechanical ventilation (MV), the use of vasoactive drugs, and renal replacement therapy (RRT).

### Outcomes

The primary outcome was 28-day mortality. Secondary outcomes included the length of hospital and ICU stays, the occurrence of shock, respiratory failure, pneumonia, AKI, and sepsis.

### Statistical analysis

Descriptive analysis included all participants. Continuous variables were presented as mean ± standard deviation (SD) for normally distributed data or as median and interquartile range (IQR) for skewed distributions. To compare differences between groups, categorical variables were analyzed using the chi-squared test. For continuous variables, the Student’s t-test was employed for normal distributions, whereas the Mann–Whitney U test was used for skewed distributions.

PSM was utilized to equate baseline characteristics, employing a 1:1 nearest neighbor matching approach with a caliper width of 0.05. The standardized mean difference (SMD) was used to evaluate the effectiveness of the matching process, with an SMD of less than 0.1 deemed acceptable. Multivariable Cox regression and Kaplan–Meier survival curves were employed to examine the association between β-blocker use and 28-day mortality.

Sensitivity analysis was performed on specific subgroups, such as patients without CHF and hypertension, to evaluate the impact of β-blocker therapy on 28-day mortality. This analysis assessed the differential benefits of various β-blockers, including Metoprolol, and considered the effects of different administration routes (oral and intravenous) to provide a comprehensive understanding of the overall efficacy of β-blocker therapy. Subgroup analyses were performed to determine the consistency of the impact of β-blocker therapy on 28-day mortality across different subgroups, including age, gender, CHF, hypertension, diabetes, renal disease, and GCS score. The study variables had less than 5% missing data, as detailed in Supplementary Table S1. Missing values were imputed using the ‘mice’ package in R software. The ‘mice’ method was chosen for its ability to handle both continuous and categorical variables effectively. It generates multiple imputed datasets, providing more reliable estimates and better accounting for uncertainty compared to single imputation methods.

All statistical analyses were conducted using R software (version 4.4.1) and Free Statistics software version 2.0. A p-value below 0.05 was deemed to indicate statistical significance.

## Results

### Basic characteristics

**FIGURE 2:**
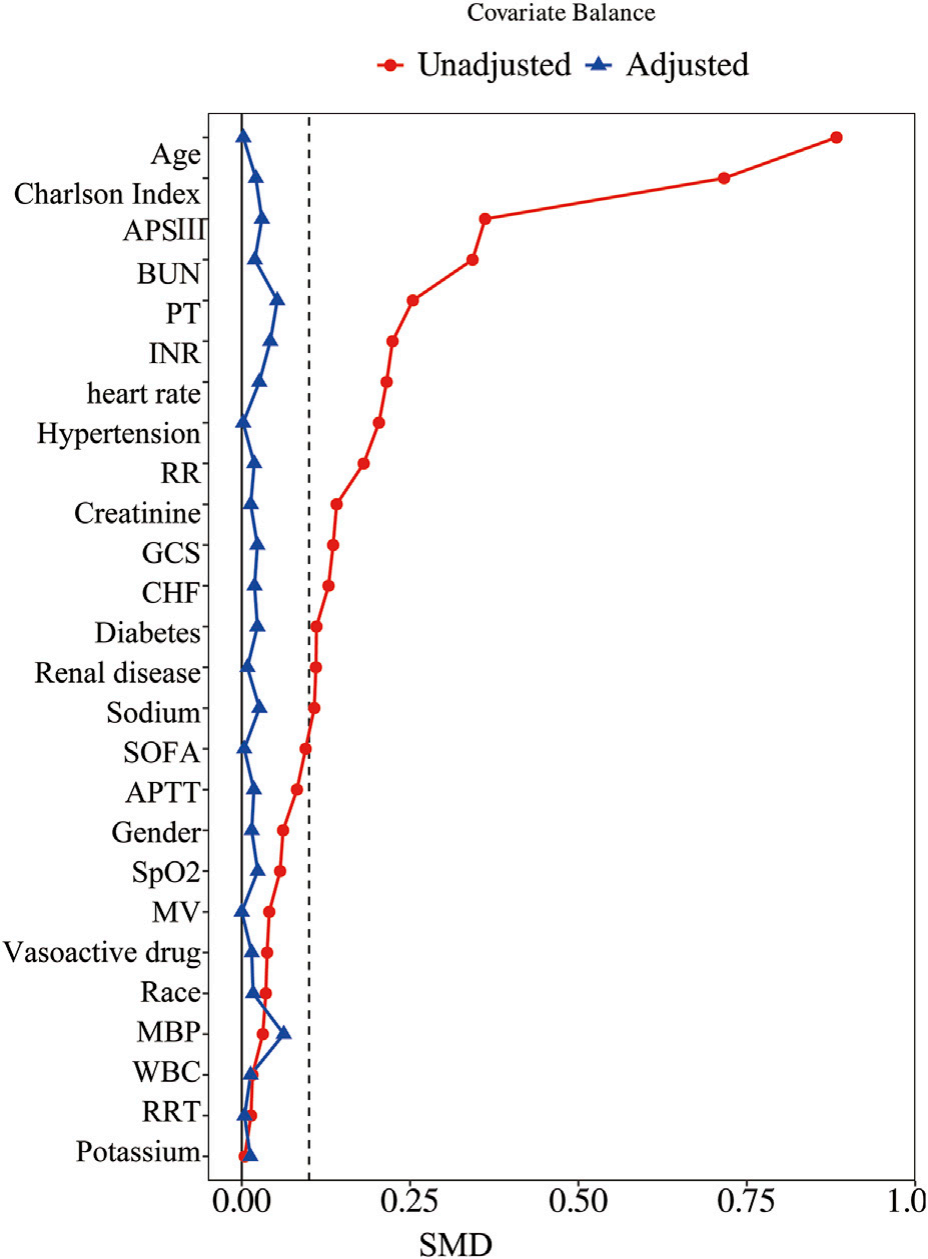
Standardized mean difference of variables before and after propensity score matching. SMD, standardized mean difference; APSIII, Acute Physiology Score III; BUN, blood urea nitrogen; PT, prothrombin time; INR, international normalized ratio; RR, respiratory rate; GCS, Glasgow Coma Scale; CHF, chronic heart failure; SOFA, Sequential Organ Failure Assessment; APTT, activated partial thromboplastin time; SpO2, blood oxygen saturation; MV, mechanical ventilation; MBP, mean blood pressure; WBC, white blood cells; RRT, renal replacement therapy.

Before PSM, 1,516 patients were analyzed, with 750 in the β-blocker group and 766 in the non-β-blocker group. Metoprolol was the most frequently used β-blocker (41.75% of patients), followed by Atenolol (6.00%) and Propranolol (2.31%) (Supplementary Table S2). Compared with the non-β-blocker group, the β-blocker group had older patients (median age: 79 vs. 63 years; P < 0.001) and a higher proportion of females (42.27% vs. 35.77%; P = 0.010). Additionally, the β-blocker group had significantly higher rates of comorbidities, including CHF, hypertension, diabetes, and renal disease (all P < 0.001). These patients also had higher Charlson Index, SOFA, and APS III scores, as well as higher BUN and creatinine levels (all P < 0.01). After PSM, 473 matched pairs of patients were obtained, with baseline characteristics well-balanced between the two groups (Table 1; Figure 2).

**TABLE 1:** Baseline characteristics.

Variables	Before PSM					After PSM				
	Total (n = 1,516)	Non-β-blocker (n = 766)	β-blocker (n = 750)	P value	SMD	Total (n = 946)	Non-β-blocker (n = 473)	β-blocker (n = 473)	P value	SMD
Age (year)	72 (55, 84)	63 (44, 78)	79 (66, 86)	<0.001	0.882	73 (60, 84)	73 (59, 84)	74 (60, 83)	0.930	0.002
Gender, n (%)				0.010					0.643	
Female	591 (38.98)	274 (35.77)	317 (42.27)		0.132	383 (40.49)	195 (41.23)	188 (39.75)		0.030
Male	925 (61.02)	492 (64.23)	433 (57.73)		0.132	563 (59.51)	278 (58.77)	285 (60.25)		0.030
Race, n (%)				0.152					0.579	
Other	479 (31.6)	255 (33.29)	224 (29.87)		0.075	308 (32.56)	150 (31.71)	158 (33.40)		0.036
White	1,037 (68.4)	511 (66.71)	526 (70.13)		0.075	638 (67.44)	323 (68.29)	315 (66.60)		0.036
Heart rate (beats/min)	79 (70, 91)	77 (69, 88)	81 (71, 93)	<0.001	0.219	78 (69, 90)	79 (70, 89)	78 (69, 90)	0.522	0.026
MBP (mmHg)	81 (75, 89)	82 (75, 89)	81 (75, 88.50)	0.697	0.031	82 (75, 89)	82 (75, 89)	82 (75, 88)	0.549	0.064
Respiratory rate (beats/min)	18 (16, 20)	18 (16, 20)	18 (17, 20)	<0.001	0.181	18 (16, 20)	18 (16, 20)	18 (16, 20)	0.818	0.019
SpO2 (%)	97.40 ± 1.83	97.44 ± 1.87	97.35 ± 1.78	0.350	0.049	97.33 ± 1.85	97.31 ± 1.93	97.35 ± 1.77	0.726	0.024
WBC (K/uL)	8.6 (6.6, 11.1)	8.7 (6.5, 11.3)	8.5 (6.7, 11.0)	0.457	0.015	8.5 (6.4, 10.8)	8.6 (6.3, 10.9)	8.4 (6.5, 10.8)	0.777	0.012
BUN (mg/dL)	14 (10, 20)	12 (8, 17)	16 (12, 22.75)	<0.001	0.343	15 (11, 20)	15 (10, 19)	15 (11, 20)	0.379	0.022
Creatinine (mg/dL)	0.8 (0.6, 1.0)	0.8 (0.6, 0.9)	0.8 (0.7, 1.1)	<0.001	0.147	0.8 (0.6, 1.0)	0.8 (0.6, 1.0)	0.8 (0.6, 1.0)	0.766	0.013
Sodium (mEq/L)	137.79 ± 4.88	138.05 ± 4.92	137.52 ± 4.83	0.036	0.109	137.66 ± 4.91	137.72 ± 5.10	137.60 ± 4.72	0.691	0.027
Potassium (mEq/L)	3.8 (3.5, 4.2)	3.8 (3.5, 4.1)	3.8 (3.5, 4.2)	0.388	0.007	3.8 (3.5, 4.2)	3.8 (3.5, 4.2)	3.8 (3.5, 4.2)	0.334	0.013
INR	1.1 (1.0, 1.2)	1.1 (1.0, 1.2)	1.1 (1.0, 1.2)	<0.001	0.223	1.1 (1.0, 1.2)	1.1 (1.0, 1.2)	1.1 (1.0, 1.2)	0.214	0.044
PT (s)	12.2 (11.3, 13.4)	12.0 (11.2, 13.0)	12.5 (11.5, 13.7)	<0.001	0.256	12.2 (11.3, 13.4)	12.1 (11.2, 13.4)	12.3 (11.5, 13.5)	0.121	0.055
APTT (s)	26.8 (24.6, 29.4)	26.7 (24.6, 29.0)	26.9 (24.7, 29.6)	0.167	0.078	26.6 (24.6, 29.4)	26.6 (24.6, 29.5)	26.6 (24.6, 29.4)	0.788	0.016
CHF, n (%)	193 (12.73)	48 (6.27)	145 (19.33)	<0.001	0.331	105 (11.1)	48 (10.15)	57 (12.05)	0.352	0.058
Hypertension, n (%)	698 (46.04)	276 (36.03)	422 (56.27)	<0.001	0.408	507 (53.59)	254 (53.70)	253 (53.49)	0.948	0.004
Diabetes, n (%)	337 (22.23)	128 (16.71)	209 (27.87)	<0.001	0.249	245 (25.9)	117 (24.74)	128 (27.06)	0.414	0.052
Renal Disease, n (%)	185 (12.2)	51 (6.66)	134 (17.87)	<0.001	0.293	106 (11.21)	51 (10.78)	55 (11.63)	0.680	0.026
Charlson Index	4 (2, 5)	2 (0, 5)	5 (3, 6)	<0.001	0.715	4 (2, 5)	4 (2, 5)	4 (2, 5)	0.620	0.021
GCS	14 (12, 15)	14 (13, 15)	14 (12, 15)	0.003	0.141	14 (12, 15)	14 (12, 15)	14 (12, 15)	0.639	0.024
SOFA	3 (2, 5)	3 (2, 4)	3 (2, 5)	0.005	0.093	3 (2, 5)	3 (2, 4)	3 (2, 5)	0.462	0.004
APSⅢ	34 (26, 44)	32 (23, 41)	37 (29, 48)	<0.001	0.357	35 (27, 45)	35 (26, 45)	35 (27, 45)	0.247	0.032
MV, n (%)	401 (26.45)	218 (28.46)	183 (24.40)	0.073	0.095	248 (26.22)	124 (26.22)	124 (26.22)	1.000	0.000
Vasoactive Drug, n (%)	222 (14.64)	98 (12.79)	124 (16.53)	0.039	0.101	147 (15.54)	77 (16.28)	70 (14.80)	0.530	0.042
RRT, n (%)	37 (2.44)	13 (1.70)	24 (3.20)	0.058	0.085	24 (2.54)	13 (2.75)	11 (2.33)	0.679	0.028

Abbreviation: MBP, mean blood pressure; SpO2, blood oxygen saturation; WBC, white blood cells; BUN, blood urea nitrogen; INR, international normalized ratio; PT, prothrombin time; APTT, activated partial thromboplastin time; CHF, chronic heart failure; GCS, glasgow coma scale; SOFA, sequential organ failure assessment; APS III, Acute Physiology Score III; MV, mechanical ventilation; RRT, renal replacement therapy.

### Primary outcome

**FIGURE 3:**
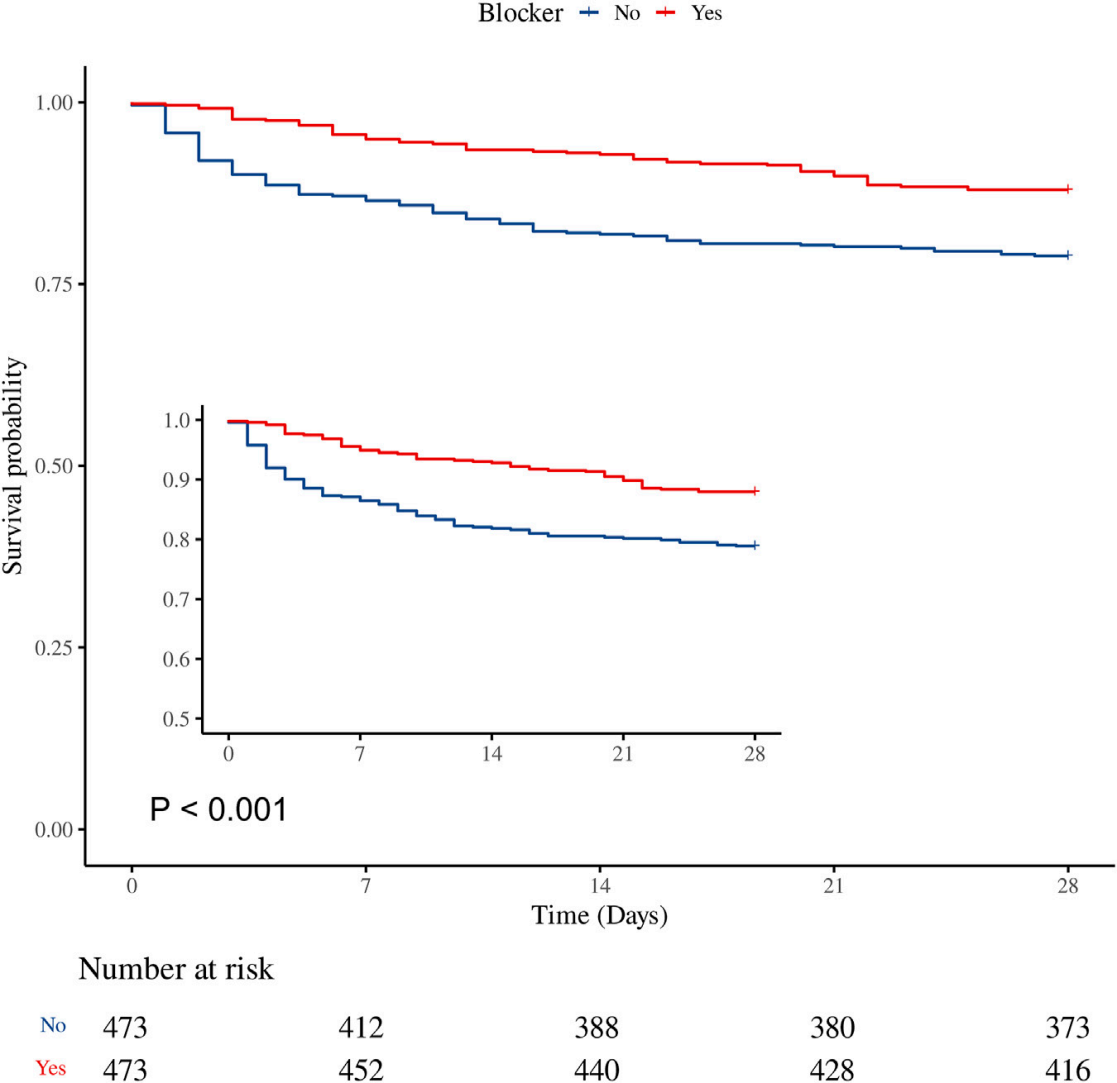
Kaplan–Meier survival curve for 28-day mortality. β-blocker use was associated with improved 28-day survival in the matched cohort (HR 0.53, 95% CI 0.38–0.73, P < 0.001).

The overall 28-day mortality rate was 16.60% (157/946). The β-blocker group had a significantly lower 28-day mortality rate compared with the non-β-blocker group (12.05% vs. 21.14%, P < 0.001; Table 2). Supplementary Table S3 shows that β-blocker use improved 28-day survival in the GCS 3-8 and GCS 13–15 groups (P = 0.010 and P < 0.001, respectively), but had no effect in the GCS 9–12 (moderate) group (P = 0.861). Supplementary Table S4 summarizes baseline characteristics by 28-day survival status, and Supplementary Table S5 provides univariate analysis results for variables with significant differences. After PSM, the multivariable Cox regression analysis revealed that β-blocker use was associated with a significant reduction in 28-day mortality (HR 0.43, 95% CI: 0.31–0.60, P < 0.001). Consistent results were observed using inverse probability weighting (IPW; HR 0.42, 95% CI: 0.29–0.60, P < 0.001). Kaplan–Meier survival curves further demonstrated improved survival in the β-blocker group (Figure 3).

**TABLE 2:** Association between β-blocker use and primary outcome in the crude analysis, multivariable analysis, and propensity score analysis.

Analysis	28-day mortality	P value
The primary outcome		
No. of death/no. of patients (%)		
Total	157 (16.60)	
β-blocker	57/473 (12.05)	
Non-β-blocker	100/473 (21.14)	
Univariable analysis^a^	1.13 (0.88–1.45)	0.325
Multivariable analysis^b^	0.59 (0.08–4.51)	0.608
Propensity-score analyses		
Univariable analysis^c^	0.53 (0.38–0.73)	<0.001
Multivariable analysis^d^	0.43 (0.31–0.60)	<0.001
With IPW^e^	0.42 (0.29–0.60)	<0.001

^a^ Univariate COX, before PSM.

^b^ A multivariate COX, model adjusted for confounders before PSM.

^c^ Univariate COX, after PSM.

^d^ A multivariate COX, model adjusted for confounders after PSM.

^e^ A multivariate COX, model adjusted for confounders with inverse probability weighting according to the propensity score.

Confounding variables are those listed in Supplementary Table S4 that have a P value of less than 0.05. HR, hazard ratio; PSM, propensity score matching; IPW, inverse probability weighting.

### Sensitivity analysis

Sensitivity analyses confirmed the robustness of the findings. β-blocker use significantly reduced 28-day mortality in patients without CHF and hypertension (HR 0.35, 95% CI: 0.20–0.61, P < 0.001). Both Metoprolol (HR 0.49, 95% CI: 0.34–0.68, P < 0.001) and other β-blockers (HR 0.26, 95% CI: 0.07–0.91, P = 0.036) were associated with reduced mortality. Similarly, both oral (HR 0.39, 95% CI: 0.25–0.61, P < 0.001) and intravenous (HR 0.48, 95% CI: 0.31–0.75, P = 0.001) routes of administration demonstrated significant survival benefits (Table 3).

**TABLE 3:** Sensitivity analysis of the relationship between β-blockers use and 28-day mortality.

Variable	Crude HR (95%CI)	P value	Adjusted HR (95%CI)^a^	P value
Non-CHF and Non- hypertension	0.46 (0.28–0.78)	0.003	0.35 (0.20–0.61)	<0.001
Metoprolol	0.64 (0.46–0.89)	0.008	0.49 (0.34–0.68)	<0.001
Other type β-blockers	0.21 (0.07–0.66)	0.007	0.26 (0.07–0.91)	0.0036
Oral administration	0.41 (0.27–0.61)	<0.001	0.39 (0.25–0.61)	<0.001
Intravenous administration	0.77 (0.50–1.16)	0.212	0.48 (0.31–0.75)	0.001

^a^ Multivariate COX, model adjusted for confounders.

Confounding variables are those listed in Supplementary Table S4 that have a P value of less than 0.05. HR, hazard ratio; 95% CI, 95% confidence interval.

### Secondary outcomes

The β-blocker group had significantly longer hospital stays (7.89 vs. 5.45 days, P < 0.001) and ICU stays (2.94 vs. 2.33 days, P < 0.001). After adjustment, β-blocker use remained independently associated with longer hospital (adjusted β 4.73, 95% CI: 3.42–6.05, P < 0.001) and ICU stays (adjusted β 1.62, 95% CI: 1.06–2.18, P < 0.001). No significant differences were observed between the two groups for shock (P = 1.00), respiratory failure (P = 0.283), pneumonia (P = 0.064), AKI (P = 0.052), or sepsis (P = 0.841) (Table 4).

**TABLE 4:** Analysis of length of stay and clinical complications.

Analysis	Non-β blockers (n = 473)	β-blockers (n = 473)	P value	Adjusted coefficient (95% CI)	P value
Length of Stay					
Lenth of hospital stay	5.45 (3.57,9.13)	7.89 (5.18, 14.49)	<0.001	4.73 (3.42–6.05)^a^	<0.001
Length of ICU stay	2.33 (1.63,3.99)	2.94 (1.78, 5.75)	<0.001	1.62 (1.06–2.18)^a^	<0.001
Complications					
Shock	13 (2.75)	13 (2.75)	1.00	1.07 (0.40–2.90)^b^	0.890
Respiratory failure	68 (14.38)	80 (16.91)	0.283	1.44 (0.96–2.17)^b^	0.080
Pneumonia	16 (3.38)	28 (5.92)	0.064	1.87 (0.98–3.56)^b^	0.056
AKI	305 (64.48)	333 (70.40)	0.052	1.32 (0.99–1.74)^b^	0.056
Sepsis	180 (38.05)	183 (38.69)	0.841	1.04 (0.79–1.39)^b^	0.763

^a^ Linear regression model was used to evaluate the association between β-blocker use and the length of hospital and ICU, stay, adjusting for confounding variables.

^b^ Logistic regression model was employed to estimate the impact of β-blocker use on clinical complications, adjusting for confounding variables.

Confounding variables are those listed in Supplementary Table S4 that have a P value of less than 0.05. HR, hazard ratio; 95% CI, 95% confidence interval.

### Subgroup analysis

**FIGURE 4:**
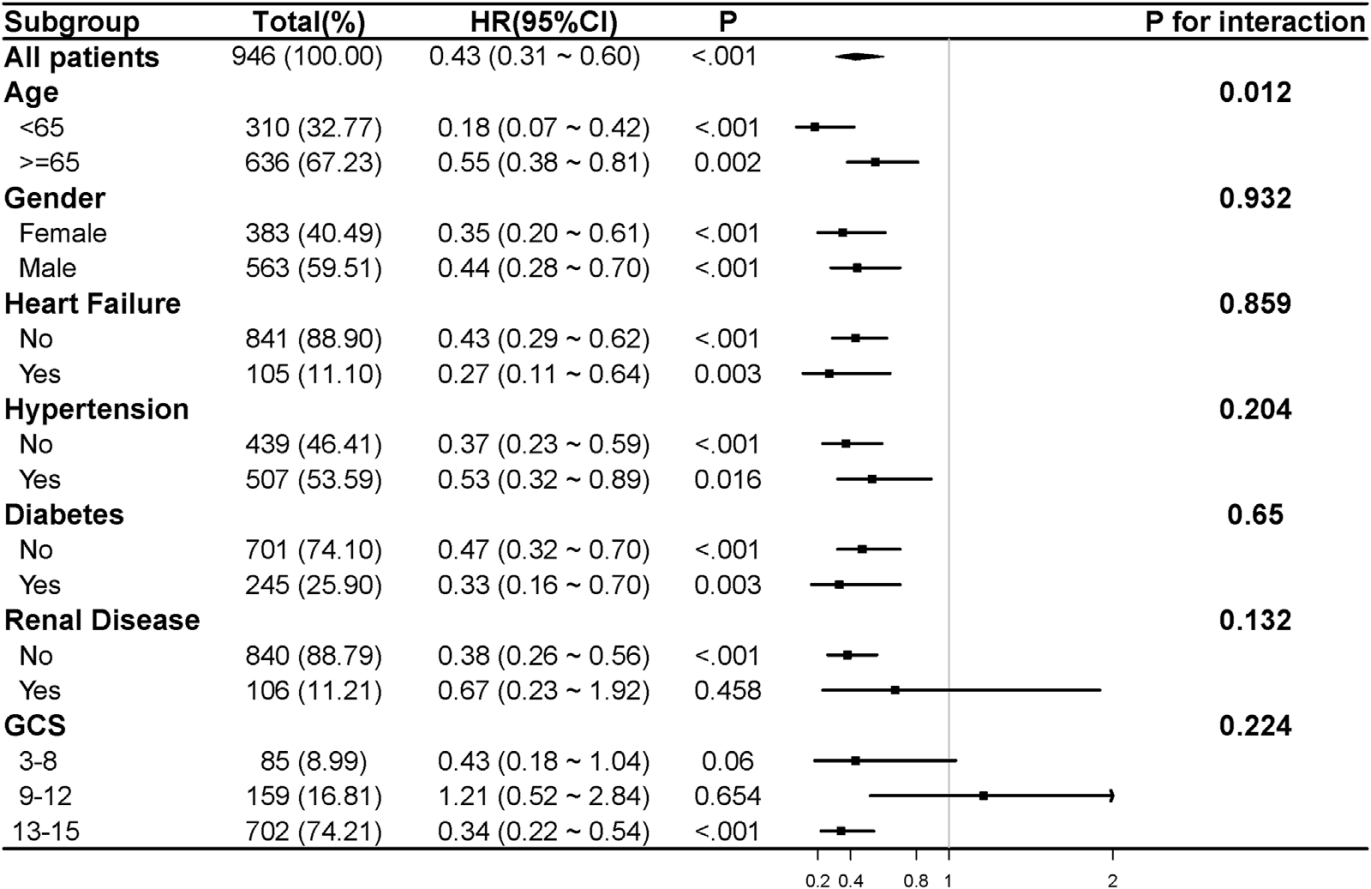
The association between β-blocker use and 28-day mortality in various subgroups. HR and 95% CI were adjusted for confounding variables. Confounding variables included those listed in Supplementary Table S4 with a P value of less than 0.05. CHF, chronic heart failure; GCS, Glasgow Coma Scale; HR, hazard ratio; 95% CI, 95% confidence interval.

Subgroup analysis (Figure 4) demonstrated that β-blocker use was associated with reduced 28-day mortality across most subgroups (HR 0.43, 95% CI: 0.31–0.60, P < 0.001). The survival benefit was more pronounced in patients aged <65 years (HR 0.18, 95% CI: 0.07–0.42, P < 0.001) than in those aged ≥65 years (HR 0.55, 95% CI: 0.38–0.81, P = 0.002). β-blocker therapy showed consistent benefits across subgroups based on gender, CHF, hypertension, and diabetes. However, no significant association was observed in patients with renal disease (HR 0.67, 95% CI: 0.23–1.92, P = 0.458) or in those with moderate TBI severity (GCS 9–12: HR 1.21, 95% CI: 0.52–2.84, P = 0.654).

## Discussion

In this large retrospective cohort study, 1,516 patients were analyzed, with 750 receiving β-blocker therapy and 766 not receiving it. After PSM, 473 pairs of patients were matched. The findings demonstrated that β-blocker therapy was associated with improved 28-day mortality. Secondary outcomes revealed that β-blocker use was linked to longer hospital and ICU stays, while no significant differences were observed for complications such as shock, respiratory failure, pneumonia, AKI, and sepsis.

The primary objective in managing TBI is to avert secondary injury, which refers to the physiological worsening of the initial trauma. This involves strategies to stabilize the patient and minimize further damage to brain tissues, thus improving overall outcomes. When TBI occurs, there is a significant release of catecholamines, including adrenaline, noradrenaline, and dopamine, which are critical in the body’s stress response (Rizoli et al., 2017). Elevated catecholamine levels can elevate heart rate and blood pressure and lead to cardiac arrhythmias, which in turn can worsen cerebral perfusion pressure and exacerbate brain injury (Neil-Dwyer et al., 1990). Catecholamines also lead to hyperglycemia and a higher metabolic rate, impairing neuronal recovery (Sherwin et al., 1980). Additionally, they cause excitotoxicity and neuroinflammation, contributing to secondary brain injury and worsening outcomes. These neurotransmitters interfere with the regulation of cerebral blood flow and compromise the integrity of the blood-brain barrier (BBB). This disruption allows neurotoxic substances to penetrate the brain, thereby exacerbating the injury further (Claassen et al., 2021).

β-blockers, commonly prescribed for cardiovascular conditions have demonstrated neuroprotective properties in diverse experimental models and preclinical studies. These agents can modulate the sympathetic nervous system’s response, potentially reducing the harmful effects of excessive catecholamine release following TBI. The efficacy of β-blockers in patients with TBI has been extensively investigated, with substantial evidence indicating a beneficial effect on mortality rates. Ahl et al. demonstrated that beta-blocker therapy significantly reduced mortality and improved long-term functional outcomes, likely through the modulation of sympathetic hyperactivity (Ahl et al., 2017). Similarly, Zangbar et al. observed that metoprolol reduced mortality (21% versus 32%; P = 0.04) independently of heart rate control, suggesting that the benefits extend beyond cardiovascular stabilization, potentially by attenuating the post-injury catecholamine surge (Zangbar et al., 2016). Mohseni et al. further supported these findings, reporting that beta-blockers were associated with a significant reduction in in-hospital mortality (Mohseni et al., 2015). Cotton et al. corroborated these observations, noting a 71% reduction in mortality risk among TBI patients treated with beta-blockers (Cotton et al., 2007). However, while these studies highlight immediate survival benefits, they largely overlook broader clinical outcomes, such as the duration of hospital and ICU stays. Our research addresses this gap by leveraging the comprehensive MIMIC-IV database and applying sophisticated propensity score matching to assess not only survival but also the impact of beta-blocker therapy on hospitalization lengths and the incidence of complications in a diverse patient cohort. Our findings reveal that while beta-blockers improve survival rates, they also tend to extend the duration of hospital and ICU stays. Moreover, our study hints at possible associations with complications such as pneumonia and AKI, though these observations did not reach statistical significance, indicating a need for cautious interpretation and further investigation.

Several mechanisms have been proposed to explain the potential benefits of β-blockers in the management of TBI. A primary mechanism involves attenuating the hyperadrenergic catecholamine state accompanying TBI, which can exacerbate penumbral neuroinflammation and increase BBB permeability. Studies have shown that β-blockers like propranolol can dose-dependently reduce leukocyte mobilization and BBB permeability, thereby mitigating cerebral edema and enhancing neurological recover (Lopez et al., 2022). Additionally, β-blockers mitigate the risk of venous thromboembolism (VTE) in TBI patients, suggesting a role in modulating associated coagulopathy during the catecholamine surge (Dhillon et al., 2021). Their metabolic and immunomodulatory effects, including reducing sympathetic activation, hypermetabolism, and modulating glucose homeostasis and cytokine expression, further support their therapeutic utility in TBI management (Loftus et al., 2016). The neurocardiac axis theory and the occurrence of neurogenic stunned myocardium offer additional understanding of β-blockers’ involvement in brain-heart interactions in TBI. They help maintain optimal mean arterial pressure and cerebral perfusion pressure, potentially improving clinical outcomes (El-Menyar et al., 2017). Moreover, β-blockers have been observed to mitigate posttraumatic hyperthermia (PTH) by reducing febrile episodes’ frequency, increasing intervals between episodes, and limiting maximum temperature rises, with propranolol demonstrating notable efficacy in this context (Asmar et al., 2021).

Our study suggests that β-blocker use is linked to extended hospital and ICU stays in patients with TBI. This relationship has been examined in various studies, yielding mixed results. For instance, a retrospective cohort study found no significant impact of β-blocker use on hospital stay in TBI patients, though the study highlighted considerable variability in the dosage and timing of β-blocker administration (Kelly-Hedrick et al., 2023). Conversely, a meta-analysis indicated that patients with severe TBI who received β-blockers experienced a significantly extended hospital stay (17.30 vs. 11.02 days) compared to those who did not receive β-blockers, although the increase in ICU stay (9.00 vs. 6.84 days) was not statistically significant (Zagales et al., 2023). Another systematic review revealed that β-blockers were associated with a higher incidence of cardiopulmonary and infectious complications, potentially leading to longer hospital and ICU stays (Hart et al., 2023). However, a matched case-control study discovered that patients treated with esmolol had a shorter hospital stay (18.0 vs. 26.8 days, P < 0.01) compared to the control group. It is important to note that this study had a small sample size, so the findings should be interpreted with caution (Ahl et al., 2017). In our study, the extended hospital and ICU stays observed in the β-blocker group may partially be explained by trends in clinical complications, including higher rates of pneumonia (5.92% vs. 3.38%, P = 0.064) and AKI (70.40% vs. 64.48%, P = 0.052). Although these differences did not reach statistical significance, they suggest a potential relationship that warrants further exploration. Overall, while β-blockers appear to offer clear benefits in reducing mortality in TBI patients, their impact on hospital and ICU stays remains variable. Further high-quality randomized trials are needed to clarify these relationships and guide their integration into clinical practice.

Subgroup analysis indicates that the protective benefits of β-blockers are more evident in patients younger than 65 years, showing a substantial reduction in HR (0.18, P < 0.001) and a significant interaction p-value for age (P = 0.012). This suggests that younger patients may derive greater benefit from β-blocker therapy, potentially due to better physiological resilience and fewer comorbidities compared to older patients. The age-specific efficacy highlights the importance of considering patient demographics when tailoring TBI treatment strategies. Our study found that β-blockers did not significantly improve outcomes in patients with renal disease (HR 0.67, P = 0.458). This absence of significant benefit may be attributed to multiple physiological and pharmacokinetic factors. Renal dysfunction is known to alter the pharmacokinetics of β-blockers, leading to prolonged drug half-life and potential accumulation, which could diminish the protective effects or introduce adverse effects. Furthermore, the pathophysiological interplay between renal disease and TBI may exacerbate systemic inflammation, oxidative stress, and hemodynamic instability, potentially counteracting the neuroprotective benefits of β-blockers. Additionally, in patients with moderate TBI (GCS 9–12), β-blocker therapy did not show the expected benefits in reducing 28-day mortality. This observation may be attributed to several factors. Firstly, the relatively low mortality rate in the GCS 9–12 group, with only 26 deaths out of 159 patients, limits the statistical power to detect a significant effect. The wide confidence interval (HR 1.21, 95% CI: 0.52–2.84) suggests substantial uncertainty in the estimate of the treatment effect, indicating that random error may have contributed to the lack of observed benefit. Secondly, the pathophysiology of TBI differs between mild, moderate, and severe injuries, particularly in terms of sympathetic nervous system activity. β-blockers reduce sympathetic overactivity and catecholamine levels, which are typically elevated in more severely injured patients. In the GCS 9–12 group, the sympathetic nervous system may be less activated compared to those with severe TBI (GCS 3–8), thus limiting the potential therapeutic effect of β-blockers. In contrast, the higher baseline sympathetic nervous system activity in the severe TBI group may have allowed for a more pronounced effect of β-blockers. Lastly, the larger sample size in the mild TBI group (GCS 13–15), with 702 patients, provided greater statistical power to detect a significant reduction in 28-day mortality, even though the physiological mechanisms of injury may be less severe. Taken together, the low mortality rate, small sample size, and relatively lower sympathetic nervous system activation in the moderate TBI group likely limited the detection of any significant benefit from β-blocker therapy. Further studies with larger cohorts and a more refined understanding of the physiological mechanisms in different severity levels of TBI are needed to clarify the role of β-blockers in this patient population.

This study has several limitations that warrant consideration. First, the age distribution of the cohort was skewed toward older adults, which may limit the generalizability of the findings to younger TBI populations who often present with different injury mechanisms, physiological responses, and baseline health conditions. Future research should include a more balanced age distribution to better evaluate the age-specific effects of β-blocker therapy. Second, the study population was exclusively derived from ICU admissions, which represents a more severe subset of TBI cases. This selection may not reflect the full spectrum of TBI severity or the broader clinical scenarios encountered in non-ICU settings, such as general wards or outpatient care. As such, the findings may not be directly applicable to patients with less severe TBI. Studies incorporating a broader range of TBI patients across various care settings are needed to establish the external validity of these results. Third, although PSM was employed to adjust for potential confounding factors, residual confounding cannot be completely excluded. Differences in injury severity, such as the presence of extracranial injuries, may introduce systemic inflammatory responses or hemodynamic instability, potentially influencing the efficacy of β-blocker therapy. Furthermore, variations in trauma mechanisms, such as falls versus motor vehicle collisions, may contribute to heterogeneity in clinical outcomes. Concurrent medications, including sedatives or anticoagulants, and pre-existing conditions, such as hypertension, diabetes, and renal dysfunction, could also modify the effects of β-blockers or independently affect outcomes. These factors highlight the complexity of interpreting results in this population. Fourth, the study focused on β-blocker use within the first 3 days after ICU admission, which was intended to target the acute phase of TBI when sympathetic hyperactivity and catecholamine surges are most pronounced. However, this definition of exposure may exclude potential benefits or risks of β-blockers initiated beyond this timeframe, particularly in the subacute or chronic phases of TBI. Patients who receive β-blockers after the acute phase might differ in terms of baseline characteristics, injury progression, or clinical management strategies, which could influence outcomes. Future research should explore the timing of β-blocker initiation across different phases of TBI to better understand how the timing of therapy affects clinical outcomes and whether there are benefits to extended β-blocker use beyond the acute phase.

## Conclusion

β-blocker therapy is associated with improved short-term outcomes in patients with TBI, particularly in those with mild (GCS 13–15) and severe (GCS 3–8) TBI. However, no significant benefit was observed in patients with moderate TBI (GCS 9–12). This therapy may also prolong hospital and ICU stays. Further research, including prospective studies, is warranted to uncover the mechanisms underlying these effects and to evaluate their potential integration into clinical guidelines and protocols for TBI management.

## Data Availability

The datasets presented in this study can be found in online repositories. The names of the repository/repositories and accession number(s) can be found below: https://mimic.mit.edu/.
