# Supplementary material for: Effect of β-blocker on clinical outcomes in patients with traumatic brain injury: a retrospective propensity-matched study
Source: Front Pharmacol. 2025 Jan 22;16:1465657. doi: 10.3389/fphar.2025.1465657 (PMC11794503; doi:10.3389/fphar.2025.1465657)
Supplement: Supplementary file 1 [file Table1.docx]

**Supplementary information**

Table S1 Missing number of variables.

Table S2 Details regarding the classification of β-blockers.

Table S3 Relationship between β-blocker use and 28-day survival in different GCS subgroup.

Table S4 Baseline characteristics of the 28-day mortality and 28-day survival group.

Table S5 Univariate cox proportional analysis for 28-day mortality.

Table S1 Missing number of variables.

| **Variable** | **Missing number (%)** |
| --- | --- |
| Age | 0 |
| Gender | 0 |
| Race | 0 |
| Heart rate | 3 (0.20) |
| MBP | 3 (0.20) |
| Respiratory rate | 10 (0.66) |
| SpO2 | 3 (0.20) |
| WBC | 12 (0.79) |
| BUN | 11 (0.73) |
| Creatinine | 10 (0.66) |
| Sodium | 12 (0.79) |
| Potassium | 12 (0.79) |
| INR | 70 (4.62) |
| PT | 70 (4.62) |
| APTT | 72 (4.62) |
| CHF | 0 |
| Hypertension | 0 |
| Diabetes | 0 |
| Renal Disease | 0 |
| Charlson Index | 0 |
| GCS | 3 (0.20) |
| SOFA | 0 |
| APSⅢ | 0 |
| MV | 0 |
| Vasoactive Drug | 0 |
| RRT | 0 |

Abbreviation: MBP, mean blood pressure; SpO2, blood oxygen saturation; WBC, white blood cells; BUN, blood urea nitrogen; INR, international normalized ratio; PT, prothrombin time; APTT, activated partial thromboplastin time; CHF, chronic heart failure; GCS, Glasgow Coma Scale; SOFA, Sequential Organ Failure Assessment; APSIII, Acute Physiology Score III; MV, mechanical ventilation; RRT, renal replacement therapy.

Table S2 Details regarding the classification of β-blockers.

| β-Blockers | Patients(%) |
| --- | --- |
| Metoprolol | 663/1516 (41.75) |
| Atenolol | 91/1516 (6.00) |
| Propranolol | 35/1516 (2.31) |
| Esmolol | 21/1516 (1.39)) |
| Nadolol | 9/1516 (0.59) |
| Bisoprolol | 2/1516 (0.13) |
| Betaxolol | 1/1516 (0.07) |
| Acebutolol | 1/1516 (0.07) |

Table S3 Relationship between β-blocker use and 28-day survival in different GCS subgroup.

| **TBI** | **Total** | **Non-β-blockers** | **β-blockers** | **P value** |
| --- | --- | --- | --- | --- |
| GCS 3-8 |  |  |  | 0.010 |
| 28-day survival | 51 (60.00) | 17 (44.74) | 34 (72.34) |  |
| 28-day mortality | 34 (40.00) | 21 (55.26) | 13 (27.66) |  |
| GCS 9-12 |  |  |  | 0.861 |
| 28-day survival | 133 (83.65) | 69 (84.15) | 64 (83.12) |  |
| 28-day mortality | 26 (16.35) | 13 (15.85) | 13 (16.88) |  |
| CGS 13-15 |  |  |  | <0.001 |
| 28-day survival | 605 (86.18) | 287 (81.30) | 318 (91.12) |  |
| 28-day mortality | 97 (13.82) | 66 (18.70) | 31 (8.88) |  |

Table S4 Baseline characteristics of the 28-day mortality and 28-day survival group.

| **Variables** | **Total**  **(n = 946)** | **28-day survival**  **(n = 789)** | **28-day mortality**  **(n = 157)** | **P value** |
| --- | --- | --- | --- | --- |
| Age (year) | 73 (60, 84) | 72 (59, 83) | 80 (66, 88) | <.001 |
| Gender, n (%) |  |  |  | 0.430 |
| Female | 383 (40.49) | 315 (39.92) | 68 (43.31) |  |
| Male | 563 (59.51) | 474 (60.08) | 89 (56.69) |  |
| Race, n (%) |  |  |  | <.001 |
| Other | 308 (32.56) | 238 (30.16) | 70 (44.59) |  |
| White | 638 (67.44) | 551 (69.84) | 87 (55.41) |  |
| Heart rate(beats/min) | 78 (69, 90) | 78 (69, 89) | 80 (72, 94) | 0.028 |
| MBP (mmHg) | 82 (75, 89) | 82 (75, 89) | 80 (73, 87) | 0.011 |
| Respiratory rate(beats/min) | 18 (16, 20) | 18 (16, 20) | 18 (17, 21) | 0.012 |
| SpO2 (%) | 97.33 ± 1.85 | 97.20 ± 1.71 | 97.97 ± 2.36 | <.001 |
| WBC (K/uL) | 8.5 (6.4, 10.8) | 8.20 (6.3, 10.4) | 10.5 (7.7, 13.1) | <.001 |
| BUN (mg/dL) | 15 (11, 20) | 14 (10, 19) | 17 (12, 25) | <.001 |
| Creatinine (mg/dL) | 0.8 (0.6, 1.0) | 0.8 (0.6, 1.0) | 0.9 (0.7, 1.2) | 0.002 |
| Sodium (mEq/L) | 138 (135.25, 141) | 138 (135, 141) | 139 (136, 142) | 0.019 |
| Potassium (mEq/L) | 3.8 (3.5, 4.2) | 3.8 (3.5, 4.2) | 3.8 (3.4, 4.2) | 0.648 |
| INR | 1.1 (1.0, 1.2) | 1.1 (1.0, 1.2) | 1.2 (1.1, 1.3) | <.001 |
| PT (s) | 12.2 (11.3, 13.4) | 12.1 (11.2, 13.3) | 12.7 (11.7, 13.9) | <.001 |
| APTT (s) | 26.6 (24.6, 29.4) | 26.8 (24.6, 29.4) | 26.1 (24.1, 29.5) | 0.423 |
| CHF, n (%) | 105 (11.10) | 77 (9.76) | 28 (17.83) | 0.003 |
| Hypertension, n (%) | 507 (53.59) | 434 (55.01) | 73 (46.50) | 0.051 |
| Diabetes, n (%) | 245 (25.90) | 203 (25.73) | 42 (26.75) | 0.789 |
| Renal Disease, n (%) | 106 (11.21) | 85 (10.77) | 21 (13.38) | 0.345 |
| Charlson Index | 4 (2, 5) | 4 (2, 5) | 5 (3, 6) | <.001 |
| GCS | 14 (12, 15) | 14 (13, 15) | 14 (9, 15) | 0.710 |
| SOFA | 3 (2, 5) | 3 (2, 4) | 5 (3, 7) | <.001 |
| APSⅢ | 35 (27, 45) | 34 (26, 43) | 44 (34, 57) | <.001 |
| MV, n (%) | 248 (26.22) | 147 (18.63) | 101 (64.33) | <.001 |
| Vasoactive Drug, n (%) | 147 (15.54) | 85 (10.77) | 62 (39.49) | <.001 |
| RRT, n (%) | 24 (2.54) | 18 (2.28) | 6 (3.82) | 0.399 |

Abbreviation: MBP, mean blood pressure; SpO2, blood oxygen saturation; WBC, white blood cells; BUN, blood urea nitrogen; INR, international normalized ratio; PT, prothrombin time; APTT, activated partial thromboplastin time; CHF, chronic heart failure; GCS, Glasgow Coma Scale; SOFA, Sequential Organ Failure Assessment; APS III, Acute Physiology Score III; MV, mechanical ventilation; RRT, renal replacement therapy.

Table S5 Univariate cox proportional analysis for 28-day mortality.

| **Variables** | **HR (95%CI)** | **P value** |
| --- | --- | --- |
|  |  |  |
| Age | 1.02 (1.01 - 1.03) | 0.001 |
| Race |  |  |
| Other | 1.00 (Reference) |  |
| White | 0.56 (0.41 - 0.76) | <0.001 |
| Heart Rate | 1.02 (1.01 - 1.03) | <0.001 |
| MBP | 0.98 (0.96 - 0.99) | 0.008 |
| Respiratory Rate | 1.10 (1.04 - 1.16) | <0.001 |
| SpO2 | 1.28 (1.16 - 1.42) | <0.001 |
| WBC | 1.02 (1.01 - 1.03) | <0.001 |
| BUN | 1.02 (1.01 - 1.03) | <0.001 |
| Creatinine | 1.12 (1.00 - 1.26) | 0.053 |
| Sodium | 1.05 (1.01 - 1.09) | 0.019 |
| INR | 4.64 (2.55 - 8.47) | <0.001 |
| PT | 1.15 (1.08 - 1.22) | <0.001 |
| CHF |  |  |
| No | 1.00 (Reference) |  |
| Yes | 1.83 (1.21 - 2.75) | 0.004 |
| MV |  |  |
| No | 1.00 (Reference) |  |
| Yes | 6.51 (4.69 - 9.04) | <0.001 |
| Vasoactive Drug |  |  |
| No | 1.00 (Reference) |  |
| Yes | 4.48 (3.25 - 6.17) | <0.001 |
| Blocker |  |  |
| No | 1.00 (Reference) |  |
| Yes | 0.53 (0.38 - 0.73) | <0.001 |

Abbreviation: MBP, mean blood pressure; SpO2, blood oxygen saturation; WBC, white blood cells; BUN, blood urea nitrogen; INR, international normalized ratio; PT, prothrombin time; CHF, chronic heart failure; MV, mechanical ventilation.
